# Predicting mortality in patients with non-ischemic dilative cardiomyopathy: Potential of extracellular volume imaging by cardiovascular magnetic resonance

**DOI:** 10.1186/1532-429X-16-S1-P88

**Published:** 2014-01-16

**Authors:** Katharina Koopmann, Ulf K Radunski, Sebastian Bohnen, Lukas Radziwolek, Gunnar Lund, Christian Stehning, Gerhard Adam, Stefan Blankenberg, Kai Muellerleile

**Affiliations:** 1 Cardiology, University Heart Center, Hamburg, Germany; 2 Radiology, University Medical Center, Hamburg, Germany; 3 Philips Research, Hamburg, Germany

## Background

Predicting mortality in patients with non-ischemic dilated cardiomyopathy (NIDCM) is currently demanding and requires complex models. Assessing the extracellular volume fraction (ECV) by T1-mapping cardiovascular magnetic resonance (CMR) is an attractive approach to quantify myocardial injury as a potential predictor of adverse events in these patients. This study evaluated ECV imaging by T1-mapping CMR in comparison with the established "Seattle Heart Failure Model" (SHFM; http://www.seattleheartfailuremodel.org).

## Methods

This study included 50 patients with heart failure and reduced left ventricular (LV) ejection fraction due to non-ischemic cardiomyopathy. The SHFM was used to estimate 1, 2 and 5 year mortalities as well as life expectancy, respectively. T1 quantification was performed at 1.5 Tesla using the modified Look-Locker inversion-recovery (MOLLI) sequence on 3 short-axes before and 15 minutes after administration of 0.075 mmol/kg gadolinium-BOPTA. Global myocardial ECV was then calculated from native and post-contrast T1 maps generated by a dedicated plug-in written for the OsiriX software, respectively.

## Results

Median predicted 1-, 2- and 5-year mortalities were 4% (interquartile range 2-6%), 7% (interquartile range 5-12%) and 16% (interquartile range 12-28%) in the study population, respectively. Median estimated life expectancy was 13 years (interquartile range 9-15 years). A significant correlation was found between global myocardial ECV and predicted 1- (r = 0.47; p < 0.001), 2- (r = 0.46; p < 0.001) and 5-year mortality (r = 0.46; p < 0.001), respectively. Furthermore, a significant inverse correlation was found between global myocardial ECV and predicted life expectancy (r = -0.37; p < 0.01).

## Conclusions

Myocardial injury as quantified by ECV-imaging correlates well with predicted mortality and has great potential to improve risk stratification in patients with heart failure due to NIDCM.

## Funding

No funding.

